# Choosing interventions to eliminate forest malaria: preliminary results of two operational research studies inside Cambodian forests

**DOI:** 10.1186/s12936-020-03572-3

**Published:** 2021-01-20

**Authors:** Amber Kunkel, Chea Nguon, Sophea Iv, Srean Chhim, Dom Peov, Phanith Kong, Saorin Kim, Sarun Im, Mark Debackere, Nimol Khim, Jean Popovici, Sreynet Srun, Amélie Vantaux, Jean-Olivier Guintran, Benoit Witkowski, Patrice Piola

**Affiliations:** 1 Epidemiology and Public Health Unit, Institut Pasteur du Cambodge, Phnom Penh, Cambodia; 2 Emerging Diseases Epidemiology Unit, Institut Pasteur, Paris, France; 3 National Center for Parasitology, Entomology and Malaria Control, Phnom Penh, Cambodia; 4 Malaria Molecular Epidemiology Unit, Institut Pasteur du Cambodge, Phnom Penh, Cambodia; 5 Partners for Development, Phnom Penh, Cambodia; 6 Malaria Consortium, Phnom Penh, Cambodia; 7 World Health Organization, Cambodia Country Office, Phnom Penh, Cambodia

**Keywords:** Malaria, *Plasmodium falciparum*, *Plasmodium vivax*, Cambodia, Greater mekong subregion, Forest, Forest-goers, Rapid diagnostic test, Mass screening and treatment

## Abstract

**Background:**

Rapid elimination of *Plasmodium falciparum* malaria in Cambodia is a goal with both national and international significance. Transmission of malaria in Cambodia is limited to forest environments, and the main population at risk consists of forest-goers who rely on forest products for income or sustenance. The ideal interventions to eliminate malaria from this population are unknown.

**Methods:**

In two forested regions of Cambodia, forest-goers were trained to become forest malaria workers (FMWs). In one region, FMWs performed mass screening and treatment, focal screening and treatment, and passive case detection inside the forest. In the other region, FMWs played an observational role for the first year, to inform the choice of intervention for the second year. In both forests, FMWs collected blood samples and questionnaire data from all forest-goers they encountered. Mosquito collections were performed in each forest.

**Results:**

Malaria prevalence by PCR was high in the forest, with 2.3–5.0% positive for *P. falciparum* and 14.6–25.0% positive for *Plasmodium vivax* among forest-goers in each study site. In vectors, malaria prevalence ranged from 2.1% to 9.6%, but no *P. falciparum* was observed. Results showed poor performance of mass screening and treatment, with sensitivity of rapid diagnostic tests equal to 9.1% (95% CI 1.1%, 29.2%) for *P. falciparum* and 4.4% (95% CI 1.6%, 9.2%) for *P. vivax*. Malaria infections were observed in all demographics and throughout the studied forests, with no clear risk factors emerging.

**Conclusions:**

Malaria prevalence remains high among Cambodian forest-goers, but performance of rapid diagnostic tests is poor. More adapted strategies to this population, such as intermittent preventive treatment of forest goers, should be considered.

## Background

The development and spread of artemisinin resistant malaria in the Greater Mekong Subregion (GMS; consisting of Cambodia, Laos, Myanmar, Thailand, Vietnam, and parts of China) has made elimination of malaria from this region a global priority [1]. Previously, the emergence and spread of chloroquine and sulfadoxine-pyrimethamine resistance from this region led to major increases in malaria mortality in Africa; rapid malaria elimination from this region is considered the best way to avoid repeating the past [2, 3]. In Cambodia, the National Center for Parasitology, Entomology, and Malaria Control and partners have deployed several strategies towards this malaria elimination goal, including the implementation of a system of village malaria workers (community health workers capable of diagnosing and treating malaria within remote villages); selection of national first-line treatment based on results of treatment efficacy studies; implementation of single low-dose primaquine; and dissemination of long-lasting insecticide-treated nets [4]. Small trials of mass drug administration have been implemented in selected villages, with generally positive but short-lived results [5–7]. An increase in cases in 2017 relative to 2016 [8] led stakeholders to consider even more drastic approaches, including foci investigation and classification of local cases (similar to the Chinese 1-3-7 strategy) [9, 10].

Malaria transmission in Cambodia and throughout the GMS is concentrated in forest environments [11, 12]. However, until recently the primary malaria control strategies in Cambodia have almost all been implemented outside of forests, in either villages or health centers, which may not fully capture asymptomatic carriers from this this high-risk population. Studies of screening and treatment approaches in villages have generally shown low yield, likely because these are not the sites where transmission occurs [13, 14]. Though other studies have been conducted at rubber plantations, the populations who work on plantations are different from forest goers and the risk of malaria transmission is apparently lower than inside natural forests [15]. Forests in the GMS are harsh environments visited primarily by young men for logging and other economic activities [16]. Forest goers may be either local to the area or mobile or migrant populations [17]. Forest goers often sleep outdoors in temporary camps, limiting the utility of traditional vector control tools such as insecticide-treated nets or indoor residual spraying [18]. One approach towards malaria control inside forests is tailored vector control measures such as hammock nets, but these may be of limited utility depending on patterns of user behaviour [18–20]. Another idea that has been proposed is the implementation of chemoprophylaxis to forest goers [12]. A new system of mobile malaria workers, intended to screen (with rapid diagnostic tests, RDTs) and treat individuals both around and inside the forest fringe, has also been recently implemented [21].

The proliferation of different approaches to elimination of forest malaria raises the question of which strategies could be most effective in quelling this source of transmission in the GMS [22]. This study uses data emerging from two ongoing operational research projects inside Cambodian forests to assess the potential effectiveness of various screening, treatment, and vector control interventions inside the forest. These studies are the first to be conducted truly inside the forest, and provide a unique look at the dynamics of forest goers and malaria in this environment. The preliminary results presented here were planned analyses used to select an intervention for the second phase of one of the two studies, and could also inform the best choices of interventions for elimination of forest malaria in the GMS and beyond.

## Methods

### Study design

This study reports preliminary findings of two separate in-forest malaria control studies (Table 1). Both studies centre on the training and deployment of Forest Malaria Workers (FMWs) inside study forests. FMWs were selected from forest goers already familiar with the study forests. Each FMW is assigned to a specified area (“sector”) of the forest, where they are required to stay 5 days per week with the objective of enrolling any forest goers in that sector in the study.

**Table 1:** Overview of the two studies

	Observation-intervention study	MSAT study
Objectives	To observe the characteristics of forest goers and the malaria risk inside a selected forest (Year 1, presented here), and then select and implement an intervention to eliminate malaria there (Year 2, in progress)	To eliminate malaria in the study forest using a combination of MSAT, passive case detection, and reactive case detection
Forested Area	500 km^2^	1000 km^2^
Forest Location	North-East Cambodia (Mondulkiri and Ratanakiri provinces, near Kratie border)	North-East Cambodia (Kratie and Stung Treng provinces)
Forest selection criteria	Isolation from other forests and study sites Presence of *P falciparum*	Presence of *P falciparum*
Time period included	March 2019–December 2019 (Study continuing through February 2021)	August 2019–December 2019 (Study continuing through December 2020)
Interventions applied	No intervention; except small amounts of ACTs and RDTs given to FMWs in case of urgent need	MSAT Passive case detection Reactive case detection
Number of sectors (1 FMW per ssector)	30	36
Median (IQR) sector size	15.2 (13.5, 15.6) km^2^	25.9 (24.8, 27.0) km^2^

**Fig. 1:**
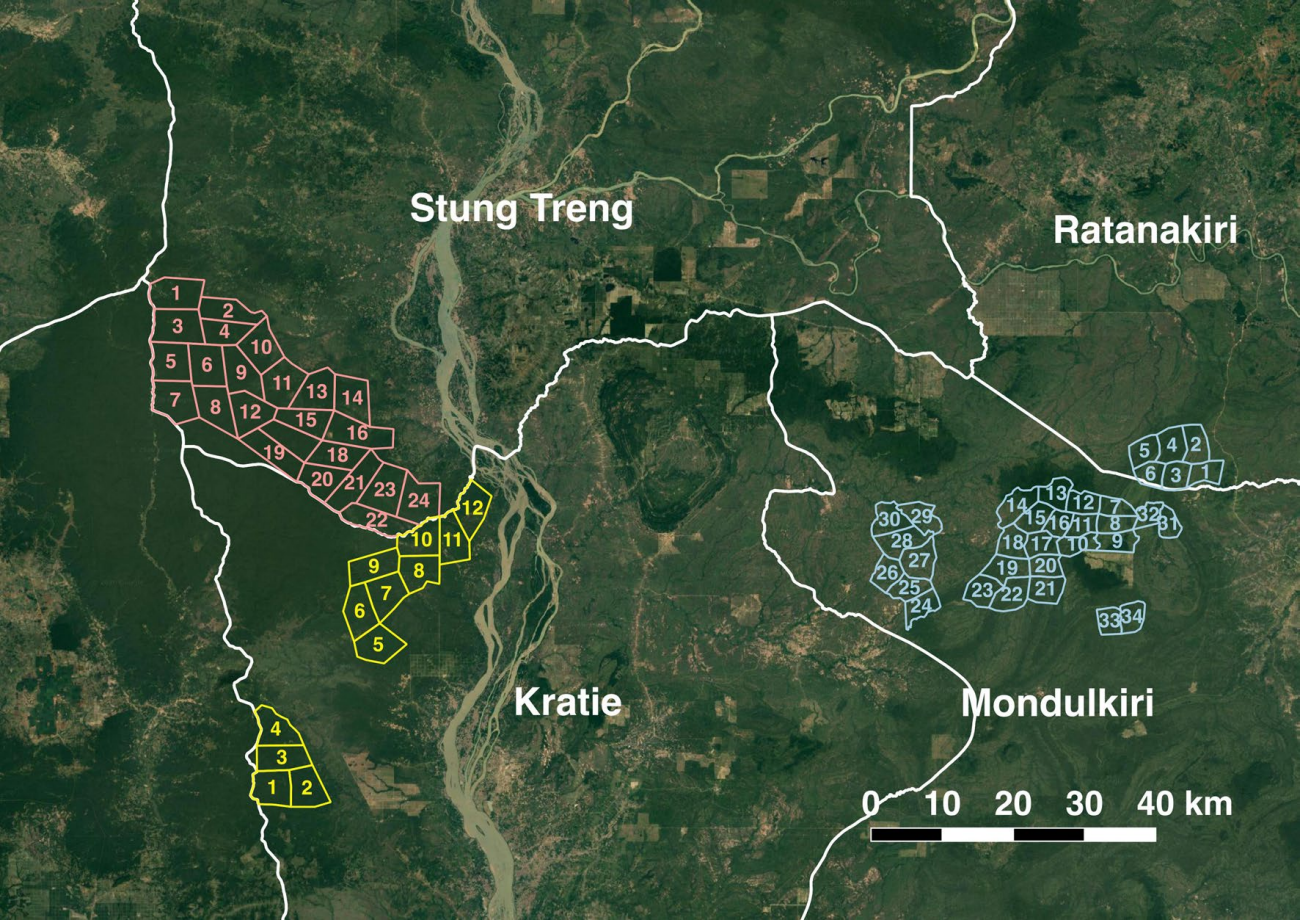
Map of study sectors. Blue sectors are those in the observation-intervention study. Pink (Stung Treng) and yellow (Kratie) sectors are those in the MSAT study. The background shows Google satellite images of the study area downloaded 29 June 2020

The first study is located inside a small forested area (approximately 500 km^2^) in Mondulkiri and Ratanakiri provinces, near the Kratie province border (Fig. 1). This forest was selected based on satellite data and field visits due to its size, the presence of *Plasmodium falciparum* malaria recorded in neighbouring health centres, and its isolation from other forests. The objective of the first year of this study, which began in March 2019, was to observe the characteristics of people working inside the forest environment and their malaria risk in the absence of any intervention. All forest goers aged 10 or older who provided informed consent (including parental consent and child assent for minors < 18 years old) submitted a questionnaire and were asked to provide a dried blood spot (DBS). Forest goers who were continuously or repeatedly in the forest could be enrolled at most every 2 weeks. The data obtained from this first year was intended to be strictly observational and inform a choice of intervention for the following year, from March 2020–March 2021. Due to ethical concerns, however, FMWs were provided with small supplies of malaria RDTs (SD Bioline Malaria Ag P.f/P.v) and artesunate-mefloquine (AS-MQ) (Artesunate/Mefloquine 100mg/200mg tablets, Cipla, the first-line malaria treatment in Cambodia) during the observation year in case they encountered any sick forest goers unable to return to the nearest health centre for treatment. The final outcomes of this study will be based on a comparison of the observation and intervention years, both within the forest and in neighboring health centers and village malaria workers. This study is referred to as the “observation-intervention” study.

The second study is located in a forested area of approximately 1000 km^2^ in Kratie and Stung Treng provinces, constituting one part the Prey Lang forest, which stretches across five provinces (Fig. 1). This forest area was chosen based on its size and the presence of *P. falciparum* malaria recorded in neighbouring health centres. This study began in August 2019 in Stung Treng province and September 2019 in Kratie Province, and will continue until December 2020. Its objective was to intervene in the study forest using a combination of mass screening (with the same RDTs) and treatment (with AS-MQ, MSAT), passive case detection of febrile cases, and reactive case detection among the colleagues of any identified cases. All forest goers of any age who provided informed consent were given a questionnaire, asked to provide a DBS, and tested for malaria by RDT. Parental consent was required for minors < 18 years old, as well as assent of the minor if at least 10 years old. Forest goers who were continuously or repeatedly in the forest could be enrolled every 2 weeks. Additionally, febrile cases could seek out the FMWs for testing and treatment. In case of a positive test, FMWs were encouraged to test any forest goers who had travelled or worked in the forest with the case and not yet been tested. Positive cases were provided with treatment (ASMQ) and, beginning in November 2019, with insecticide-treated hammock nets (Deltamethrin 55mg/m^2^, Yahe). Final outcomes of this study will be based on the comparison of malaria notifications from intervention health centers surrounding the study forest and control health centers in other parts of the country. This study is referred to as the “MSAT” study.

The overall goal of both studies is to fully or nearly eliminate *P. falciparum* malaria from the study forests by the end of the study period. The results presented here are preliminary results through to Dec 31, 2019.

### Forest sectorization

The areas of the forests to be covered in these studies were chosen based on satellite imagery followed by repeated exploratory missions to the forest. Owing to the size and isolation of these forests, these exploratory missions involved spending multiple days in the forest by motorbike. Mobile phones were used to track the paths used by research staff inside the forest, and they recorded where they encountered forest goers, camps, and water sources.

In the observation-intervention study, 30 sectors were initially selected, with a median (IQR) sector size of 15.2 (13.5, 15.6) km^2^. In the MSAT study, 36 sectors were initially selected, with a median (IQR) sector size of 25.9 (24.8, 27.0) km^2^. The number of FMWs was equal to the number of sectors. However, the sectors did not all remain fixed within the study. Data from the first few months of the observation-intervention study revealed that some sectors had few forest goers, while additional exploratory missions suggested other areas that should be included. As a result, four sectors (12, 13, 20, and 21) were removed and replaced with four new ones (31, 32, 33, and 34) the week of 8 July 2019. In the MSAT study, four of the initially selected sectors had no active FMW through the time of this analysis due to difficulties in obtaining permission from a company working in those areas.

### Training and monitoring of FMWs

FMWs were provided with Android smartphones for geolocation, monitoring, and electronic data entry. A 1 week training session was conducted for FMWs from each study site on malaria awareness, the study objectives and procedures, use of the smartphones, collection of dried blood spots, and malaria case management.

Electronic data entry was done via Kobo toolbox. FMW smartphones were also equipped with the GPS Logger application to track their movements inside the forest and BackCountry Navigator to help them understand the boundaries of their sector. FMWs were provided with powerbanks to maintain smartphone power inside the forest. At the end of each work week, the FMWs returned the smartphones and powerbanks to study staff, who uploaded the data collected that week and charged the devices before returning them to the FMWs. Weekly feedback on each FMW was automatically produced based on their questionnaire results and GPS tracks, and was used to improve their performance.

### Questionnaires

The questionnaires for the two studies were very similar and included questions on their activities and shelter inside the forest, their knowledge of malaria and malaria prevention methods, and their healthcare-seeking behaviour. Questions about identifying information such as name, village of residence, age, and phone number were also asked, to allow for better identification of repeat forest goers both for purposes of analysis and intervention (i.e. eventual follow-up of PCR positive cases in the MSAT study). In the observation-intervention study, forest goers were asked if they thought they had a fever on the day of the interview; those who said no were assumed to be healthy, and axillary temperatures were measured among those who said yes. In the MSAT study, axillary temperatures were measured for all participants. Those participants with temperatures > 37.5C were classified as having a fever at the time of the interview. Testing and treatment was offered to any individuals in either study presenting with a fever. Temperatures measured outside a biologically plausible range were discarded and considered as missing.

### Laboratory testing

Dried blood spots were collected from forest goers for malaria real-time PCR screening and speciation. Parasite DNA was extracted using Instagene matrix resin (Biorad, Singapore).

Screening and speciation of malaria infection was performed by targeting sequence coding for cytochrome b according to Canier et al. [23]. Cycle threshold (Ct) values were used to estimate parasites densities.

Beginning in January 2020, after the end of the results presented here, study staff have attempted to contact individuals in the MSAT study who tested positive by PCR to receive malaria treatment. Individuals testing positive by PCR in the observation-intervention study were not re-contacted for treatment.

### Entomology

Exposure to malaria vectors was determined by carrying out mosquito collection using odour-baited double net traps (BNTs as in [24]) baited with human odours and/or cow odours (observation-intervention study). The BNTs consisted of an outer untreated bed net (200 × 200 × 250 cm for the human BNT—and 300 × 300 × 300 cm for the cow BNT) from which each corner was raised 20 cm above ground and a smaller untreated inner bed net (180 × 180 × 250 cm for the human BNT and 280 × 280 × 280 cm for the cow BNT). An image is provided in the Appendix (Additional file 1: Fig S2). Mosquitoes trapped between the two nets were retrieved every hour for 10 min using a mouth aspirator. Two human volunteers rested on hammocks and were protected from mosquito bites. The BNTs were operated for 24 h for 3 days in each collection sites. For the MSAT study a before and after intervention collections were planned, whereas monthly collections were carried out in the observation-intervention study. For the MSAT project, collection sites were set up in 14 sectors and 1 village in Stung Treng province in August 2019 and in 8 sectors in Kratie province in December 2019. In the observation-intervention study, collection sites were first set up in two neighbouring villages and in 6 sectors, then the number of collection sites was increased to 3 neighbouring villages and 10 sectors in December 2019. Trap locations were sometimes moved according to the project constraints (including change of sectors, accessibility limitation during the rainy season etc.). In both study, mosquitoes were transferred to plastic cups covered with netting and labelled by date, location and hour of collection. All *Anopheles* sp. mosquitoes were morphologically identified using a taxonomic key [25]. Following morphological identification, samples were preserved at −20 ℃ until molecular biology analyses. The mosquito heads and thoraces were crushed individually and DNA was extracted using the QIAamp DNA Mini kit^®^, (Qiagen,Germany), according to the manufacturer’s instructions. A two-step semi quantitative real-time PCR was performed to detect malaria parasites, as previously described [26].

### Statistical analysis

The interim analyses presented here were intended to assist in the choice of interventions for Year 2 of the “observation-intervention” study. All analyses were made in R. The main questions and analyses are as follows.

### Characteristics of forest goers

Basic descriptions of forest goer demographics, activity patterns, and familiarity with malaria are reported as means and percentages. To describe forest goer movement patterns, it was necessary to identify repeat forest goers inside the database. The details of how this matching was done are reported in the Additional file 1: Appendix.

### Mass preventive treatment

To assess the risk-benefit profile of mass preventive treatment to forest goers, the yield and the number of people that would need to be treated (NNT) per *P. falciparum* infection and per *P. vivax* infection were calculated. The yield was calculated as number infected divided by number tested. The NNT was calculated as the inverse of the yield.

### RDT-based treatment

To determine whether RDTs should be used to assign treatment in Year 2 of the observation-intervention phase, the sensitivity and specificity of RDTs (compared to PCR) were calculated from the MSAT study. RDT readings made by FMWs were compared to those made by study staff based on photos of the RDTs uploaded into Kobo Toolbox. All RDT photos were read by at least one study staff member. The RDT photos of all individuals who tested positive for malaria (*P. falciparum* or *P. vivax*) by PCR, or whose RDTs were considered positive by the FMW or any study staff member, were read by three different staff members and the majority opinion was used.

### Risk factor-based treatment

Additional analyses examined whether any risk factors from the questionnaire could be used instead of or in addition to RDTs to target individuals for treatment. Risk factors were evaluated according to the following criteria: strength of the relationship with *P. falciparum* infection (to allow discrimination between people with and without infection); prevalence of the risk factor among individuals with *P. falciparum* infection (to ensure high intervention coverage); and consistency between the two studies (to increase generalizability of the results, and limit the possibility for spurious associations produced by multiple testing). Associations between questionnaire variables (independent variables) and *P. falciparum* PCR positivity (dependent variable) were used to assess strength of the relationship. Odds ratios and p-values were obtained from sequential univariate logistic regression models.

These analyses emphasized primarily those variables that have been identified as risk factors in studies outside of the forest, namely: age, gender, logging, history of malaria treatment, staying overnight in the forest, and having fever in the last month [18, 27–29]. The remaining variables from the questionnaire were also screened for any variables that showed strong association with *P. falciparum* malaria infection (odds ratio > 1.5) and that were present in a majority (> 50%) of *P. falciparum* infections in the output of both studies. Cut-offs based on odds ratios rather than p-values were used, as they provide measures of the magnitude of effect rather than statistical significance (which may be affected by other factors such as sample size); a high odds ratio implies high separation of risk between the different risk groups. An odds ratio of 1.5 implies only a 50% higher odds of infection inside the risk group compared to outside, and was considered the minimum acceptable value for this initial screen.

Finally, the analyses searched for geographic hotspots of *P. falciparum* infection in both study forests. Those individuals PCR positive for *P. falciparum* were considered as “cases” and those PCR negative for *P. falciparum* as “controls.” The R package sparr function “risk” was used to estimate the relative risk between the case and control 2D kernel density estimates, and considered as hotspots those regions identified as significantly higher risk (p < 0.05) [30].

### Provision of individual vector control kits, e.g. insecticide-treated hammocks

To determine the potential impact of vector control kits, the proportion of *Anopheles* mosquitoes with daytime biting behaviour was considered, and the previous use of various vector control strategies among forest goers (incl. insecticide-treated hammocks) with and without *P. falciparum* infection was compared.

### Ethical approval

The observational year of the “observation-intervention” study was approved by the Cambodia National Ethics Committee for Health Research on 19 October 2018 with study number 281 (“Blocking malaria transmission in forest vulnerable populations through forest malaria workers: a key for malaria elimination in Cambodia”). It was renewed on 6 December 2019 under study number 306. The MSAT study was approved by the Cambodia National Ethics Committee for Health Research on 28 June 2019 with study number 148 (“Comparison of effectiveness of forest-based malaria control projects in large forests of Cambodia”).

## Results

In the “observation-intervention” study, 1696 forest goer encounters, of which 1612 (95.0%) provided informed consent and completed the questionnaire, were recorded from 11 March to 31 December 2019. In the MSAT study, 1145 (99.6%) of 1150 forest goers in Stung Treng from 27 July to 31 December 2019 provided informed consent and completed the questionnaire and 467 (88.8%) of 526 forest goers in Kratie from 2 September to 31 December 2019.

The matching algorithm detected 5.7% of FGs in the “observation-intervention” and 3.6% of forest goers in the MSAT study as repeat interviews with participants already included. However, the applied algorithm cut-off ensured a high probability (99%) that identified pairs were true matches, at the cost of allowing many (> 50%) of matches to be missed (see Additional file 1: Appendix). A photo recognition tool is being developed to improve matches for future analyses.

### Characteristics of forest goers

In both studies, forest visits were often transient, with 43% of forest goers in both studies reporting spending at most 1 week per month in the forest. When comparing two visits by the same individual occurring > 30 days apart, the median distance between the two interviews was 2.2 km (inter-quartile range IQR 0.4 km, 5.9 km) in the observation-intervention study and 0.9 km (0.004 km, 4.0 km) in the MSAT study; 12% of such repeated interviews in the observation-intervention study and 0% in the MSAT study were > 15 km apart. Only 33% of forest goers from the observation-intervention study and 15% of forest goers from the MSAT study provided contact phone numbers. Most forest goers (52% in the observation-intervention study and 57% in the MSAT study) reported not having a mobile phone.

The main activities of forest goers differed by study site and time. Logging (45%) and gathering forest products (43%) were the most common activities reported in the observation-intervention study, whereas in the MSAT study gathering forest products was by far the most commonly reported activity (75%). The proportion of forest goers in the observation-intervention study who reported logging declined between June and September, corresponding to a period of crackdowns on illegal logging throughout Cambodia (Additional file 1: Fig S1); enrollments in the MSAT study began during this crackdown period.

Familiarity with malaria was generally high, with only 24% of forest goers in the observation-intervention study and 8% in the MSAT study responding that they had never heard of malaria before. Excluding these individuals, 56% of forest goers in the observation-intervention study said they had been treated for malaria in the last year and 25% had been treated before but longer than one year ago. In the MSAT study, 16% of forest goers reported malaria treatment in the last year, and 62% more than one year ago. Self-reported history of fever in the last month was declared by 38% of forest goers in the observation-intervention study compared to 31% in the MSAT study.

### Malaria infection prevalence

PCR results were available for 1450 of 1612 participants in the observation-intervention study, of whom 73 (5.0%) were positive for *P. falciparum* infection, 363 (25.0%) were positive for *P. vivax* infection, and 2 (0.1%) were positive for *Plasmodium malariae* infection. Of these, 10/73 (13.7%) positive for *P. falciparum* and 36/363 (9.9%) positive for *P. vivax* had out-of-range temperature values recorded. Of those remaining, 2/63 (3.2%) positive for *P. falciparum* had a measured fever at the time of the interview (one of which was a mixed *P. falciparum*/*P. vivax* infection); among those positive for *P. vivax*, including mixed, this result was 3/327 (0.9%). If all forest goers were to receive preventive treatment for malaria, these findings would correspond to a number needed to treat (NNT) of 19.9 (15.9, 25.2) individuals per *P. falciparum* infection and 4.0 (3.7, 4.4) individuals per *P. vivax* infection (Table 2).

**Table 2:** Malaria yield and number needed to treat per positive PCR infection

Study	N PCR results	Species	N PCR+	Yield* (95% CI)	Number needed to treat per PCR + (95% CI)
Observation-Intervention	1450	*P. falciparum*	73	0.05 (0.04, 0.06)	19.9 (15.9, 25.2)
		*P. viax*	363	0.25 (0.23, 0.27)	4.0 (3.7, 4.4)
MSAT	1598	*P. falciparum*	37	0.02 (0.02, 0.03)	43.2 (31.5, 61.1)
		*P. viax*	234	0.15 (0.13, 0.17)	6.8 (6.1, 7.7)

^*^Yield: proportion PCR+ (number positives/number tested)

In the MSAT study, PCR results were available for 1598 of 1612 participants. Of these, 37 (2.3%) were positive for *P. falciparum* infection, 234 (14.6%) were positive for *P. vivax* infection, and 10 (0.6%) were positive for *P. malariae* infection. Among those positive for *P. falciparum*, 3/37 (8.1%) had a measured fever at the time of the interview (two of which were mixed *P. falciparum*/*P. vivax* infections); among those positive for *P. vivax*, including mixed, this result was 6/234 (2.6%). If all forest goers were to receive preventive treatment, the NNT would be 43.2 (31.5, 61.1) per *P. falciparum* infection and 6.8 (6.1, 7.7) per *P. vivax* infection (Table 2).

### Performance of rapid diagnostic tests

Results of rapid diagnostic tests are based on the MSAT study. Of 1598 interviews with PCR results available, 297 (19%) had no RDT photo available or the photo was unreadable; 306 (19%) had photos taken too early, while the RDT was still bloody; and 995 (62%) had acceptable RDT photos.

Based on the acceptable photos only, for *P. falciparum*, there was 99.6% agreement between RDT readings by research staff and FMWs, and Cohen’s kappa was 0.60 (95% CI: 0.24, 0.96). For *P. vivax*, there was 99.8% agreement between RDT readings, and Cohen’s kappa was 0.89 (0.73, 1.0).

Table 3 shows the cross-tabulation of RDT and PCR results for both *P. falciparum* and *P. vivax*. The sensitivity of RDTs to detect *P. falciparum* infections in the forest was estimated at 9.1% (95% CI: 1.1%, 29.2%) and the specificity was 99.8% (99.3%, 100.0%). The sensitivity of RDTs to detect *P. vivax* infections was estimated at 4.4% (95% CI 1.6%, 9.2%) and the specificity was 99.8% (99.2%, 100.0%).

**Table 3:** RDT and PCR results for both *P. falciparum* and *P. vivax*

	PCR−	PCR+	Total
*P. falciparum*			
RDT−	971	20	991
RDT+	2	2	4
Total	973	22	995
*P. vivax*			
RDT−	855	132	987
RDT+	2	6	8
Total	857	138	995

The number of cycles required for PCR to reach positivity was lower for individuals who were positive also by RDT, suggesting higher parasite density. The screening cycle thresholds ranged from 22.7 to 30.5 for *P. vivax* cases that were RDT-positive; for *P. vivax* cases that were RDT-negative, the median (IQR) cycle threshold was 36.6 (34.6, 38.8). Among *P. falciparum* infections that were negative on RDT but positive for PCR, the median (IQR) cycle threshold was 37.3 (35.6, 40.4).

This suggests that poor RDT sensitivity to diagnose malaria in forest goers is the consequence of low parasite density. The screening cycle thresholds were 26.7 and 31.2 for the two *P. falciparum* cases detected by both RDT and PCR.

### Risk factors

Table 4 shows the association of six risk factors with *P falciparum* parasitaemia measured in both studies. These six risk factors were selected based on previous studies outside of the forest. None of these risk factors met the criteria of being both strong (odds ratio > 1.5) and common (present in > 50% of *P. falciparum* infections) across both studies. In addition, no other risk factors were identified that met these criteria in both studies. In the observation-intervention study, residence in Kratie as opposed to Mondulkiri or other provinces was a highly significant risk factor for *P. falciparum* infection (OR = 5.1, 95% CI 2.6–11.6, p < 0.001). In the MSAT study, residence in Kratie as opposed to Stung Treng or other provinces was not significantly associated with higher risk of *P. falciparum* infection (OR = 1.3, 95% CI 0.7–2.6, p = 0.40).

**Table 4:** Frequency of various malaria risk factors and strength of association with *Plasmodium falciparum* parasitaemia

Variable	Value	N (%)	N Pf+ (%)	Odds ratio	p-value
Observation-intervention study					
Age	18–49	1316 (82%)	60 (82%)	Ref	Ref
	< 18	160 (10%)	9 (12%)	1.15 (0.52, 2.25)	0.70
	50+	136 (8%)	4 (5%)	0.67 (0.2, 1.66)	0.44
Gender	Male	1480 (92%)	71 (97%)	Ref	Ref
	Female	132 (8%)	2 (3%)	0.3 (0.05, 0.98)	0.10
Logging	No	890 (55%)	31 (42%)	Ref	Ref
	Yes	722 (45%)	42 (58%)	1.68 (1.05, 2.72)	0.03
Fever last month	No	998 (62%)	35 (48%)	Ref	Ref
	Yes	614 (38%)	38 (52%)	1.76 (1.10, 2.83)	0.02
Usually stay overnight	No	351 (22%)	16 (22%)	Ref	Ref
	Yes	1261 (78%)	57 (78%)	1.02 (0.59, 1.86)	0.95
Treated for malaria before	Never	225 (14%)	8 (11%)	Ref	Ref
	< 1 month ago	292 (18%)	13 (18%)	1.17 (0.48, 3.01)	0.73
	< 1 year ago	402 (25%)	23 (32%)	1.52 (0.69, 3.67)	0.32
	> 1 year ago	311 (19%)	11 (15%)	0.94 (0.37, 2.47)	0.89
	Never heard of malaria	382 (24%)	18 (25%)	1.33 (0.58, 3.30)	0.51
MSAT Study					
Age	18–49	1372 (85%)	32 (86%)	Ref	Ref
	< 18	117 (7%)	1 (3%)	0.36 (0.02, 1.72)	0.32
	50+	122 (8%)	4 (11%)	1.41 (0.41, 3.62)	0.53
Gender	Male	1424 (88%)	32 (86%)	Ref	Ref
	Female	188 (12%)	5 (14%)	1.20 (0.41, 2.86)	0.71
Logging	No	1328 (82%)	29 (78%)	Ref	Ref
	Yes	284 (18%)	8 (22%)	1.29 (0.54,2.72)	0.53
Fever last month	No	1118 (69%)	26 (70%)	Ref	Ref
	Yes	494 (31%)	11 (30%)	0.96 (0.45, 1.90)	0.90
Usually Stay Overnight	No	111 (7%)	4 (11%)	Ref	Ref
	Yes	1501 (93%)	33 (89%)	0.59 (0.23,2.02)	0.34
Treated for malaria before	Never	327 (20%)	8 (22%)	Ref	Ref
	< 1 month ago	96 (6%)	2 (5%)	0.88 (0.13, 3.60)	0.88
	< 1 year ago	147 (9%)	4 (11%)	1.12 (0.29,3.62)	0.86
	> 1 year ago	913 (57%)	21 (57%)	0.95 (0.43, 2.28)	0.89
	Never heard of malaria	129 (8%)	2 (5%)	0.64 (0.10, 2.60)	0.58

**Fig 2:**
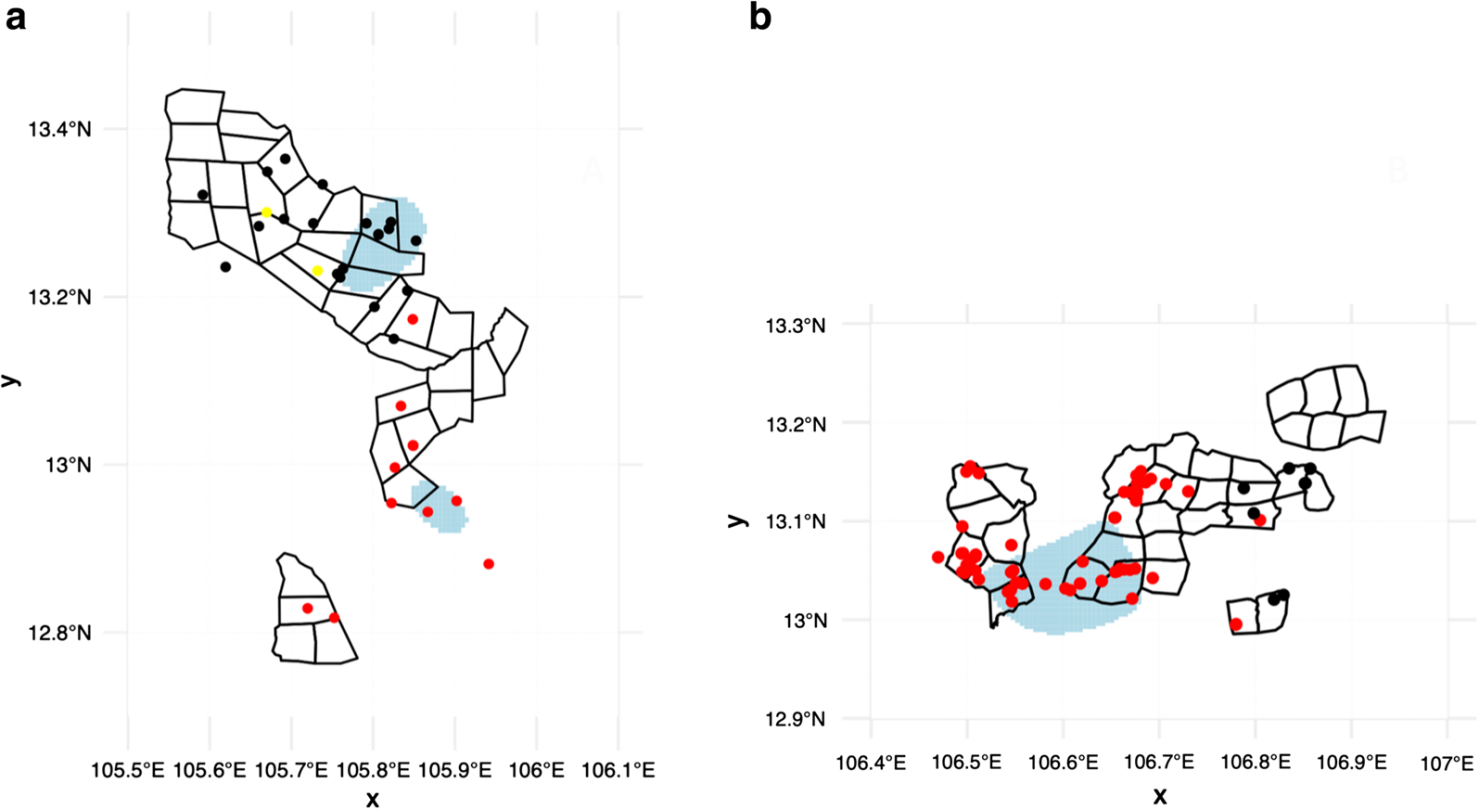
Map showing the location of all *P. falciparum* cases (points) in the MSAT study (**a**) and observation-intervention study (**b**). Blue areas indicate a significantly higher risk of *P. falciparum* infection (p < 0.05). The colours of the points indicate the residence province of the individual interviewed at that point. Red: Kratie, Black: Stung Treng (MSAT study) or Mondul Kiri (observation-intervention study), Yellow: Other.]

A hotspot area (based on human PCR positivity) was detected in the observation-interventions study area containing 33% of all *P. falciparum* infections and 17% of all interviews (Fig 2). However, *P. falciparum* infections were observed throughout in all sectors in the study area. The hotspot areas detected in the MSAT study contained 24% of *P. falciparum* infections, compared to 9% of all interviews.

### Vector population

A total of 3328 female mosquitoes were collected in the MSAT study, of which 338 were Anopheles mosquitoes. In Stung Treng province, *Anopheles dirus* represented 24% (56/234) of the collected *Anopheles* and 3 individuals were infected with malaria parasites (Additional file 1: Table S2). In addition, two Anopheles letifer mosquitoes were found infected as well (Additional file 1: Table S2). In Kratie, An. dirus represented 74% (77/104) of the collected Anopheles and 10 of them were infected by malaria parasites (Additional file 1: Table S2). Despite a low density of Anopheles mosquitoes, mosquito malaria parasite prevalence was 9.6% in Kratie and 2.1% in Stung Treng. However, in contrast to humans, no *P. falciparum* infected mosquitoes were found; mosquitoes were primarily infected with *P. vivax*, including a few infected with *P. malariae* or *Plasmodium ovale.* Infected mosquitoes were mainly collected during the early evening or early morning (Additional file 1: Table S2).

In the observation-intervention study, from March 2019 to January 2020, a total of 34,963 female mosquitoes were collected of which 7329 were *Anopheles*. Morphological identification of *Anopheles* showed a high species diversity (22 species in the 5,39 identified individuals). Three species represented more than half of the collected anopheles: *An. dirus* (23.77%), *Anopheles maculatus sensu lato* (*s.l*.) (23.78%) and *Anopheles philippinensis s.l.* (13.99%). The day biting behaviour (mosquitoes collected between 6am and 6pm) frequency was high with 22.4% of the mosquitoes biting during daytime in the human BNTs and 25% in the cow BNTS. In addition, day biting behaviour in human BNTs was usually above 20% (20.5 % to 25%) in the forest sites whereas it was below 15% in the villages (8.9%, 14.9% and 15%). At the time of the interim analyses, the overall mosquito malaria parasite prevalence was 5.1% with 68 *P. vivax* infected mosquitoes out of the 1,329 screened individuals. Infected mosquitoes were collected equally in cow BNTS (35/727) and in human BNTs (33/602). Furthermore, a high diversity of infected mosquitoes was observed with at least 12 different *Anopheles* species carrying human malaria parasites (Additional file 1: Table S2).

Additional file 1: Figures S3–S4 shows the proportions of individuals with *P. vivax* (including mixed) infections, *P. falciparum* (including mixed) infections, and no infections who said they had ever heard of or ever used individual vector control measures in each study. Although the proportions of people familiar with each vector control measure differed between study sites, they were generally similar among people with and without prevalent malaria infections.

## Discussion

The epidemiology of malaria in forest-going populations in the GMS including Cambodia is poorly characterized. This paper reports results from the first two malaria studies based *inside* the forests in Cambodia. Though the two studies are still on-going, these interim analyses were planned to assist in the determination of an intervention for the second year of the observation-intervention study.

Malaria infections were detected in both humans and mosquitoes in the study forest areas, as had been expected based on the presence of cases at nearby health centres. One of the primary conclusions is that, despite a high prevalence of malaria infection as measured by PCR, very few of these infections are detected by RDTs. The sensitivity of the RDTs in this context was very low, at 9.1% (95% CI: 1.1%, 29.2%) for *P. falciparum* and 4.4% (95% CI 1.6%, 9.3%) for *P. vivax*. Similarly poor RDT sensitivity has previously been seen at the village level in Cambodia, in a study in which 91 participants were PCR-positive for any *Plasmodium* infection, but only 2 were positive by RDT [28]. Other studies in the region have found RDT sensitivity around 40-60%, substantially higher than that seen here [31, 32]. RDT sensitivity appears to vary by setting and population, and based on the results presented here, may be particularly low inside forests.

The results suggest that a low parasite density explains the poor RDT sensitivity. Considering their occupational profile, forest goers are likely to be subjected to a chronic exposure to malaria parasites and therefore may have developed a relative immunity enabling the host to maintain a low parasite density. Previous studies have shown that submicroscopic malaria is common in the Greater Mekong Subregion, that it can last for months, and that infection density can change over time [33, 34]. Although there is no consensus on the transmissibility of individuals with submicroscopic parasites, they could represent an ongoing reservoir of infectious humans inside the receptive forest environment [33, 35, 36]. The low sensitivity of RDTs shown here suggests that RDT-based interventions such as mass screening and treatment (MSAT) are not appropriate for forest-going populations in the GMS.

Although a PCR-based MSAT (in place of RDTs) is theoretically appealing given its much higher sensitvity, this would be operationally irrelevant and is not a scalable option in Cambodia. Even in these smaller pilot projects it often took one month or longer for the dried blood spots to be transferred from the forest to the field site to the study central laboratory for testing and the results to be available. Furthermore, re-contacting forest goers after such results are available is not trivial given the small percentage who provide mobile phone numbers. Forest-goers participating in illegal logging may be particularly unwilling to provide identifying information that could allow them to be re-contacted.

A risk factor based treatment strategy could be appealing as a replacement for RDTs if an appropriate risk factor could be found. However, no risk factors were identified that were highly associated with *P. falciparum* infection and present in a majority of people with *P. falciparum* infection in both studies. Other studies have identified presence in the forest as the main risk factor for malaria infection in Cambodia [27, 28, 37]. Although the lack of strong findings could be related to sample size or misclassification bias, another possibility is that, among the population already in the forest, the risk may be more homogeneous and randomly distributed. Working in forest-specific hotspots may play some role, but the majority *P. falciparum* infections were found outside of identified hotspots; however, it was not possible to identify where these individuals were initially infected.

In terms of vector control, this study shows that *Anopheles* mosquitoes in the forest commonly bite during daytime hours, making traditional insecticide-treated nets or forest-adapted insecticide-treated hammock nets less appealing tools for this environment. Similar numbers of people infected and not infected with malaria reported ever using various vector control measures including wearing protective clothing and using repellent.

Given these identified issues, and the continued high prevalence of malaria infection inside the forest (2.3%–25.0% depending on study and malaria species), the observation-intervention study will provide preventive malaria treatment to everyone in the forest during its second year. This appears likely to be the most efficient strategy to rapidly eliminate malaria in the forest. Artemisinin-based combination therapy (ACT), using AS-MQ or dihydroartemisinin-piperaquine (DP) are appealing for this purpose as they would both eliminate existing infections and provide temporary protection from infection given the long half-life of the partner drugs. Similar to chemoprophylaxis, such “intermittent preventive treatment of forest goers” (IPTfg) would probably need to be repeated multiple times at intervals determined by the partner drug half-life, and thus has similar requirements in terms of drug safety. The two drugs with the most appealing safety and acceptability profile in Cambodia are AS-MQ and DP [12].

The problem of using AS-MQ is that it is the current first-line treatment in Cambodia, and use as IPTfg might accelerate development of resistance. However, data suggest resistance levels are currently low, and theoretical models have shown that chemoprophylaxis could in some circumstances reduce resistance in the population by reducing treatment of symptomatic cases [38, 39]; this could be the case if such deployment of AS-MQ were to substantially accelerate malaria elimination in the forest. DP is another appealing option, but experienced high failure rates in 2016 and susceptibility has not yet fully returned. Artemether-lumefrantrine was considered to have an unacceptably high pill burden for this purpose. As a result, IPTfg AS-MQ was selected for the second year of this study. Regardless of the drug selected, the risk for resistance selection implies a need to monitor trends in resistance and treat failures with second-line treatment, such as through integrated drug efficacy surveillance. Another risk associated with such an intervention is poor coverage if sufficient community buy-in is not achieved; discussions with forest goers and villagers are planned to describe the high prevalence of malaria seen in the first year of the observation-intervention study and explain the potential benefits of taking IPTfg [40, 41].

The IPTfg intervention is targeted primarily at *P. falciparum* elimination, motivated in part by widespread resistance to artemisinin in the region. However, an effect on *P. vivax* may also be observed as repeated ACT administration could offer temporary protection from *P. vivax* relapse. Nevertheless, any observed effect on *P. vivax* will likely be short-lived, and more adapted strategies such as targeted use of radical cure, should be considered secondarily for this species.

These studies were based inside forests and conducted by FMWs with strong forest knowledge but limited medical education, which led to some unique challenges. Full coverage of the population was not achieved in these studies and it is not possible to say with certainty how many forest goers were missed, as missing forest goers were not recorded systematically and may have avoided any contact with the FMWs altogether. The length of the consent process and questionnaire (about 20–30 min total) likely lowered coverage, as forest goers often work in groups and could not postpone their work to participate. Furthermore, reports of the number of people in the forest are assumed to be an underestimate, as FMWs reported that sometimes forest goers would run away when they saw them. Forest goers are often participating in illegal logging activities, which could make them suspicious of FMWs or study staff, though this risk was lowered by recruiting FMWs from the forest going community. If nonparticipation was systematic, this could affect the description of the forest goer population, but the results are still accurate for the subset of the population interviewed. The accuracy of the questionnaire responses could have been reduced if FMWs or forest goers did not accurately understand the questions. Furthermore, all risk factors were measured retrospectively, and the questionnaire did not attempt to determine the timing of past exposures such as when insecticide-treated nets were last used. However, this should be less important as the main goal was to determine associated factors that could be used for allocation of treatment, rather than elucidate causal relationships.

## Conclusions

Individuals within the forest in Cambodia have high PCR prevalence of *P. falciparum* and *P. vivax*. RDT sensitivity is low inside the forest, and the risk appears fairly homogeneous across all categories of individuals in these high-risk locations. Forest-going populations are likely to maintain a strong antimalarial immunity leading to a majority of low density infections, limiting the utility of MSAT in this environment. Given the tight deadlines to eliminate malaria in this region, intermittent preventive treatment of forest goers will be implemented inside the observation-intervention study for its intervention year. If this strategy proves feasible and effective, it could be expanded to other locations in Cambodia and in other countries attempting to eliminate forest malaria. The results suggest that the forest goer chemoprophylaxis strategies being explored by other stakeholders in Cambodia may be appropriate given the dynamics seen within these forests, but that the efficacy of hammock nets or mass testing and treatment by mobile malaria workers may be more limited, given the low sensitivity of RDTs among forest goers and the daytime biting behaviour of mosquitoes inside the forest.

## Supplementary Information

**Additional file 1: Table S1.** Specificity and sensitivity of the matching algorithm at various cutoff values. (Note: only matches with scores exceeding 0.5 were included in this validation analysis. **Fig S1.** Proportion in each study who report being in the forest for logging activities. **Fig S2.** Image of odor-baited double net trap (BNT). **Table S2.** Infected Anopheles mosquitoes collected in the two study. **Fig S3.** Proportion of individuals with *P. falciparum* infection, *P. vivax* infection, and no malaria infection (as measured by PCR) who have ever heard of or used the listed vector control measures, in the observation-intervention study. **Fig S4.** Proportion of individuals with *P. falciparum* infection, *P. vivax* infection, and no malaria infection (as measured by PCR) who have ever heard of or used the listed vector control measures, in the MSAT study.

**Additional file 2.** De-identified study database.

## Data Availability

The dataset(s) supporting the conclusions of this article is(are) included within the article (and its Additional files 1, 2). GPS coordinates are not provided out of concern for participant privacy.

## Abbreviations

ACT: Artemisinin-based combination therapy
AS-MQ: Artesunate-mefloquine
BNT: Odor-baited double net traps
Ct: Cycle threshold
DBS: Dried blood spot
DP: Dihydroartemisinin-piperaquine
FMW: Forest malaria worker
GMS: Greater Mekong Subregion
IPTfg: Intermittent preventive treatment of forest goers
IQR: Inter-quartile range
MSAT: Mass screening and treatment
NNT: Number needed to treat
PCR: Polymerase chain reaction
RDT: Rapid diagnostic test
