# Supplementary material for: Choosing interventions to eliminate forest malaria: preliminary results of two operational research studies inside Cambodian forests
Source: Malar J. 2021 Jan 20;20:51. doi: 10.1186/s12936-020-03572-3 (PMC7818569; doi:10.1186/s12936-020-03572-3)
Supplement: Supplementary file 1 — Additional file 1: Table S1. Specificity and sensitivity of the matching algorithm at various cutoff values. (Note: only matches with scores exceeding 0.5 were included in this validation analysis. Fig S1. Proportion in each study who report being in the forest for logging activities. Fig S2. Image of odor-baited double net trap (BNT). Table S2. Infected Anopheles mosquitoes collected in the two study. Fig S3. Proportion of individuals with P. falciparum infection, P. vivax infection, and no malaria infection (as measured by PCR) who have ever heard of or used the listed vector control measures, in the observation-intervention study. Fig S4. Proportion of individuals with P. falciparum infection, P. vivax infection, and no malaria infection (as measured by PCR) who have ever heard of or used the listed vector control measures, in the MSAT study. [file 12936_2020_3572_MOESM1_ESM.docx]

**SUPPLEMENTAL APPENDIX**

**Choosing interventions to eliminate forest malaria: preliminary results of two operational research studies inside Cambodian forests**

**Matching Algorithm and Results**

Matching algorithm

First, names of individuals and locations were transliterated from Khmer to approximate Latin lettering based on Unicode encodings and knowledge of the rules of the Khmer alphabet in a custom script written in R. The results were cleaned by cropping very long names (which generally consisted of repeated characters suggesting smartphone error) and recognized prefixes or suffixes.

Each new individual was then assigned a matching score for each of the individuals already present in the database before that date. For two individuals, 1 and 2, an age score, s_age_, was calculated using the equation

$$s_{age}=max\left( 0, 1-\frac{\left| {age}_{1}-{age}_{2} \right|}{20} \right)$$

Thus, s_age_=1 for two individuals of the same age, 0.95 for two individuals one year of age apart, and 0 for any individuals 20 years or more of age apart.

A name score between 0 and 1, s_name_, was calculated by comparing the two names using the R function stringsim() from package stringdist using the optimal string alignment algorithm. When first and last names were entered separately into the database, the names were concatenated. Both were directions tested and the higher score was kept, in case of confusion about where to put the family name and given name.

A village matching score was similarly calculating by comparing the village names written by two individuals using the function stringsim(). This calculation was done twice, once for birth villages (s_bv_) and one for residence villages (s_rv_).

A commune score (s_commune_) was set to equal 1 if the same commune of residence was listed by two individuals, and 0.5 otherwise. Communes were selected as multiple-choice options, not free-response strings.

A phone matching score s_phone_ was also calculated. This score was set to 0 if there was no phone number given by either participant. If phone numbers were given, they were treated as strings using the function stringsim() as above. Only those matches >90% were retained.

Early use of the algorithm found that it sometimes flagged siblings as being the same person. To limit this possibility, and noticing that siblings often worked in groups together, we a score s_dd_ = 1 if the interviews occurred on different dates, and 0 if they occurred on the same date.

Given the possibility of errors, particularly with string inputs, the final matching score s_final_ between two individuals was calculated as

$$s_{1}= s_{name}*s_{age}*s_{commune}*s_{rv}*s_{dd}$$

$$s_{2}= s_{name}*s_{age}*s_{bv}*s_{dd}$$

$$s_{3}= s_{name}*s_{age}*s_{phone}*s_{dd}$$

$$s_{final}=max(s_{1},s_{2},s_{3})$$

Because the approach to recording names and villages differed slightly between studies, as well as between sites (Stung Treng and Kratie) in the MSAT study, each matching looked only internally at the database from each study and site. We did not attempt to match individuals across studies and sites.

Assessment of matching algorithm

To assess whether the matching algorithm could accurately detect true matches, we selected a validation sample of 300 matches. We selected individual matches at random from those with matching scores of >0.5, to ensure sufficient representation of true matches. Fifty matches from the Kratie site of the MSAT study were selected, 100 from the Stung Treng site of the MSAT study, and 150 from the observation-intervention study. Photos had been taken of each individual in each study with the aim of eventually developing a facial recognition tool (still in development). The photos of the individual and the best match identified by this algorithm were compared by Khmer data managers to assess whether they appeared to be the same person or not. They then designated each of the 300 validation samples as “true matches” or “false matches.”

We calculated the area under the receiver operating characteristic curves (AUC) to assess the sensitivity and specificity of the algorithm at detecting true matches across various score thresholds for true matches. Sensitivity was defined as the probability of a true match exceeding the match score cutoff. Specificity was defined as the probability of a false match falling below the match score cutoff. Because only those samples with cutoffs >0.5 were evaluated, these estimates will tend to overestimate sensitivity in the full population and underestimate specificity.

Pooling the two study sites of the MSAT study, the estimated AUC was 0.79 (95% CI 0.71-0.86). In the observation-intervention study, the estimated AUC was 0.83 (95% CI 0.77-0.89).

Because matches were used to identify movement patterns of individuals inside the forest, we decided to prioritize having very high specificity at the cost of sensitivity. Table S1 shows the specificity and sensitivity of the matching algorithm at various score thresholds. A score threshold of 0.8 was selected for the results shown in this paper.

Table S1: Specificity and sensitivity of the matching algorithm at various cutoff values. (Note: only matches with scores exceeding 0.5 were included in this validation analysis)

|  | MSAT study | | Observation-intervention study | |
| --- | --- | --- | --- | --- |
| Score Threshold | Specificity | Sensitivity | Specificity | Sensitivity |
| 0.5 | 0 | 1 | 0 | 1 |
| 0.55 | 0.46 | 0.88 | 0.42 | 0.90 |
| 0.6 | 0.62 | 0.79 | 0.53 | 0.79 |
| 0.65 | 0.73 | 0.67 | 0.81 | 0.70 |
| 0.7 | 0.83 | 0.58 | 0.92 | 0.69 |
| 0.75 | 0.92 | 0.49 | 0.98 | 0.56 |
| 0.8 | 0.99 | 0.41 | 1.0 | 0.47 |
| 0.85 | 0.99 | 0.27 | 1.0 | 0.35 |

**Supplementary tables and figures**

Fig S1: Proportion in each study who report being in the forest for logging activities


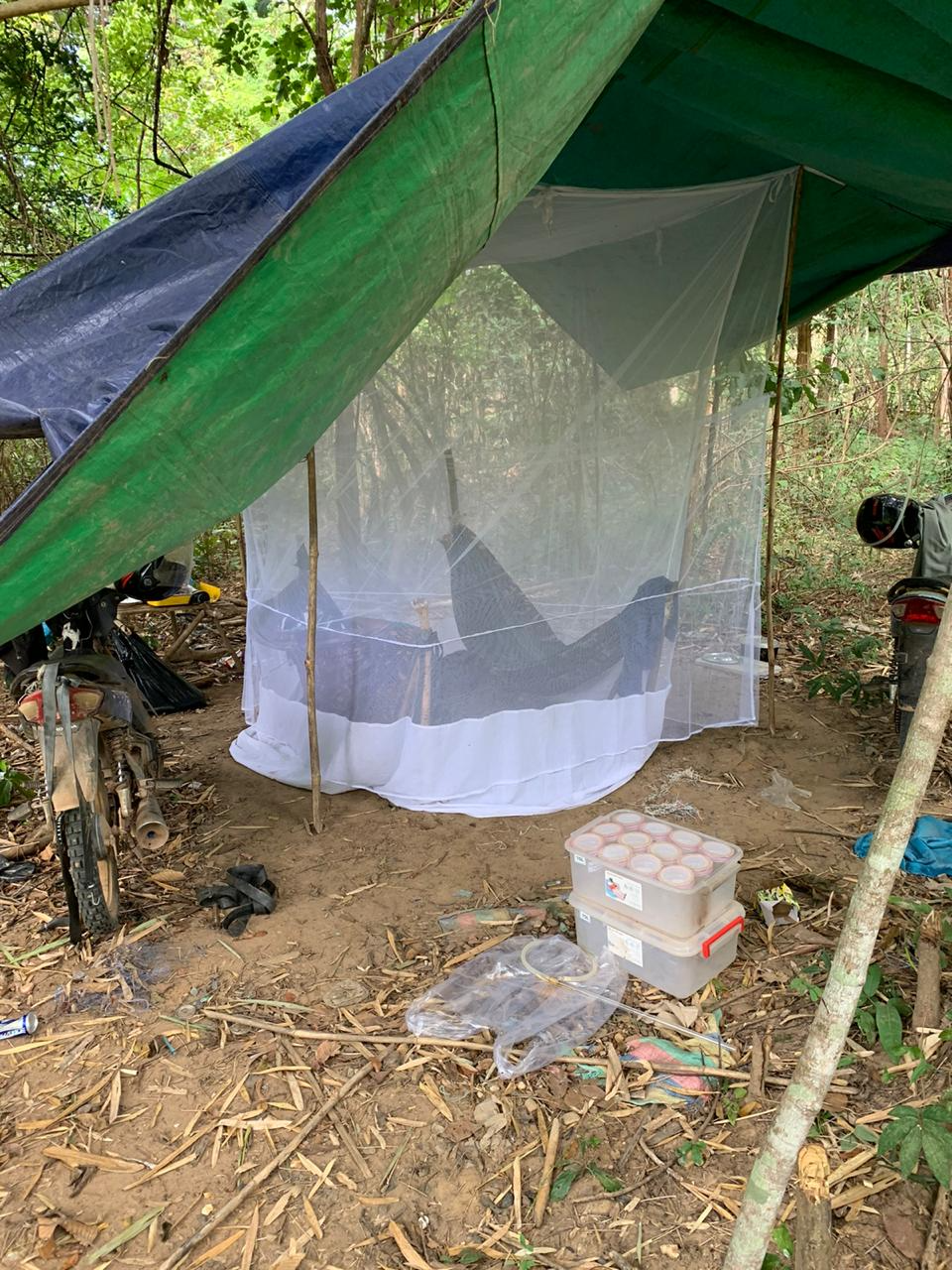


Fig S2. Image of odor-baited double net trap (BNT).

Table S2. Infected Anopheles mosquitoes collected in the two study.

| Mosquito Species | Study | Province | Sector or village | Trap type | Parasite species | Time |
| --- | --- | --- | --- | --- | --- | --- |
| *A. dirus* | MSAT | Stung Treng | S16 | Human | *P. vivax* | 9:00 PM |
| *A. dirus* | MSAT | Stung Treng | S23 | Human | *P. vivax* | 8:00 AM |
| *A. letifer* | MSAT | Stung Treng | S06 | Human | *P. vivax* | 7:00 PM |
| *A. letifer* | MSAT | Stung Treng | S17 | Human | *P. vivax* | 8:00 AM |
| *A. dirus* | MSAT | Stung Treng | S23 | Human | *P. malariae* | 2:00 AM |
| *A. dirus* | MSAT | Kratie | S04 | Human | *P. vivax* | 10:00 PM |
| *A. dirus* | MSAT | Kratie | S06 | Human | *P. malariae* | 6:00 PM |
| *A. dirus* | MSAT | Kratie | S06 | Human | *P. malariae* | 6:00 PM |
| *A. dirus* | MSAT | Kratie | S06 | Human | *P. vivax* | 6:00 PM |
| *A. dirus* | MSAT | Kratie | S06 | Human | *P. malariae* | 7:00 PM |
| *A. dirus* | MSAT | Kratie | S06 | Human | *P. vivax* & *P. ovale* | 12:00 AM |
| *A. dirus* | MSAT | Kratie | S06 | Human | *P. vivax* | 1:00 PM |
| *A. dirus* | MSAT | Kratie | S06 | Human | *P. vivax* | 1:00 PM |
| *A. dirus* | MSAT | Kratie | S07 | Human | *P. vivax* | 9:00 PM |
| *A. dirus* | MSAT | Kratie | S07 | Human | *P. vivax* | 12:00 AM |
| *A. maculatus* | Observation-Intervention | Mundolkiri | S02 | Human | *P. vivax* | NA |
| *A. barbirostris* | Observation-Intervention | Mundolkiri | S02 | Human | *P. vivax* | NA |
| *A. philippinensis* | Observation-Intervention | Mundolkiri | S02 | Human | *P. vivax* | NA |
| *A. maculatus* | Observation-Intervention | Mundolkiri | S02 | Human | *P. vivax* | NA |
| *A. philippinensis* | Observation-Intervention | Mundolkiri | S02 | Human | *P. vivax* | 6:00 PM |
| *A. barbirostris* | Observation-Intervention | Mundolkiri | S02 | Human | *P. vivax* | 4:00 AM |
| *A. philippinensis* | Observation-Intervention | Mundolkiri | S02 | Human | *P. vivax* | 8:00 AM |
| *A. jamesii* | Observation-Intervention | Mundolkiri | S02 | Human | *P. vivax* | 12:00 AM |
| *A. barbirostris* | Observation-Intervention | Mundolkiri | S02 | Human | *P. vivax* | 9:00 AM |
| *A.maculatus* | Observation-Intervention | Mundolkiri | S02 | Human | *P. vivax* | 12:00 PM |
| *A. hyrcanus* | Observation-Intervention | Mundolkiri | S02 | Human | *P. vivax* | 9:00 PM |
| *A. barbirostris* | Observation-Intervention | Mundolkiri | S02 | Cow | *P. vivax* | 9:00 PM |
| *A. barbirostris* | Observation-Intervention | Mundolkiri | S02 | Cow | *P. vivax* | 1:00 AM |
| *A. barbirostris* | Observation-Intervention | Mundolkiri | S02 | Cow | *P. vivax* | 8:00 PM |
| *A. barbirostris* | Observation-Intervention | Mundolkiri | S02 | Cow | *P. vivax* | 11:00 PM |
| *A. maculatus* | Observation-Intervention | Mundolkiri | S02 | Cow | *P. vivax* | 7:00 PM |
| *A. maculatus* | Observation-Intervention | Mundolkiri | S02 | Cow | *P. vivax* | 9:00 PM |
| *A. philippinensis* | Observation-Intervention | Mundolkiri | S02 | Cow | *P. vivax* | 2:00 PM |
| *A. barbirostris* | Observation-Intervention | Mundolkiri | S02 | Cow | *P. vivax* | 3:00 PM |
| *A. maculatus* | Observation-Intervention | Mundolkiri | S02 | Cow | *P. vivax* | 6:00 PM |
| *A. philippinensis* | Observation-Intervention | Mundolkiri | S02 | Cow | *P. vivax* | 3:00 PM |
| *A. barbirostris* | Observation-Intervention | Mundolkiri | S02 | Cow | *P. vivax* | 12:00 AM |
| Undetermined | Observation-Intervention | Mundolkiri | S02 | Cow | *P. vivax* | 2:00 AM |
| *A. barbirostris* | Observation-Intervention | Mundolkiri | S25 | Human | *P. vivax* | 1:00 AM |
| Undetermined | Observation-Intervention | Mundolkiri | S12 | Human | *P. vivax* | 3:00 AM |
| Undetermined | Observation-Intervention | Mundolkiri | S25 | Human | *P. vivax* | 2:00 PM |
| *A. barbirostris* | Observation-Intervention | Mundolkiri | S29 | Human | *P. vivax* | 1:00 AM |
| *A. dirus* | Observation-Intervention | Mundolkiri | S31 | Human | *P. vivax* | 6:00 AM |
| *A. indefinitus* | Observation-Intervention | Mundolkiri | Konva | Human | *P. vivax* | 5:00 AM |
| *A. vagus* | Observation-Intervention | Mundolkiri | Roveak | Human | *P. vivax* | 7:00 PM |
| *A. vagus* | Observation-Intervention | Mundolkiri | Roveak | Human | *P. vivax* | 9:00 PM |
| *A. maculatus* | Observation-Intervention | Mundolkiri | Roveak | Human | *P. vivax* | 12:00 AM |
| *A. philippinensis* | Observation-Intervention | Mundolkiri | Roveak | Human | *P. vivax* | 8:00 PM |
| *A. indefinitus* | Observation-Intervention | Mundolkiri | Roveak | Human | *P. vivax* | 12:00 AM |
| *A. indefinitus* | Observation-Intervention | Mundolkiri | Roveak | Human | *P. vivax* | 12:00 AM |
| *A. hyrcanus* | Observation-Intervention | Mundolkiri | Roveak | Human | *P. vivax* | 12:00 AM |
| *A. indefinitus* | Observation-Intervention | Mundolkiri | Roveak | Human | *P. vivax* | 12:00 AM |
| *A. hyrcanus* | Observation-Intervention | Mundolkiri | Roveak | Human | *P. vivax* | 12:00 AM |
| *A. indefinitus* | Observation-Intervention | Mundolkiri | Roveak | Human | *P. vivax* | 12:00 AM |
| *A. nivipes* | Observation-Intervention | Mundolkiri | Roveak | Human | *P. vivax* | 1:00 AM |
| *A. philippinensis* | Observation-Intervention | Mundolkiri | Roveak | Human | *P. vivax* | 2:00 AM |
| *A. hyrcanus* | Observation-Intervention | Mundolkiri | Roveak | Human | *P. vivax* | 4:00 AM |
| *A. philippinensis* | Observation-Intervention | Mundolkiri | Roveak | Human | *P. vivax* | 4:00 AM |
| *A. philippinensis* | Observation-Intervention | Mundolkiri | Roveak | Human | *P. vivax* | 6:00 AM |
| *A. aconitus* | Observation-Intervention | Mundolkiri | Roveak | Human | *P. vivax* | 6:00 AM |
| *A. indefinitus* | Observation-Intervention | Mundolkiri | Roveak | Cow | *P. vivax* | 8:00 PM |
| *A. indefinitus* | Observation-Intervention | Mundolkiri | Roveak | Cow | *P. vivax* | 8:00 PM |
| *A. indefinitus* | Observation-Intervention | Mundolkiri | Roveak | Cow | *P. vivax* | 8:00 PM |
| *A. indefinitus* | Observation-Intervention | Mundolkiri | Roveak | Cow | *P. vivax* | 8:00 PM |
| *A. philippinensis* | Observation-Intervention | Mundolkiri | Roveak | Cow | *P. vivax* | 1:00 AM |
| *A. indefinitus* | Observation-Intervention | Mundolkiri | Roveak | Cow | *P. vivax* | 3:00 AM |
| Undetermined | Observation-Intervention | Mundolkiri | Roveak | Cow | *P. vivax* | 3:00 AM |
| *A. indefinitus* | Observation-Intervention | Mundolkiri | Roveak | Cow | *P. vivax* | 3:00 AM |
| *A. subpictus* | Observation-Intervention | Mundolkiri | Roveak | Cow | *P. vivax* | 3:00 AM |
| *A. indefinitus* | Observation-Intervention | Mundolkiri | Roveak | Cow | *P. vivax* | 6:00 AM |
| *A. culicifacies* | Observation-Intervention | Mundolkiri | Roveak | Cow | *P. vivax* | 9:00 AM |
| *A. philippinensis* | Observation-Intervention | Mundolkiri | Roveak | Cow | *P. vivax* | 8:00 AM |
| *A. indefinitus* | Observation-Intervention | Mundolkiri | Roveak | Cow | *P. vivax* | 9:00 PM |
| *A. barbirostris* | Observation-Intervention | Mundolkiri | Roveak | Cow | *P. vivax* | 11:00 PM |
| *A. indefinitus* | Observation-Intervention | Mundolkiri | Roveak | Cow | *P. vivax* | 11:00 PM |
| *A. indefinitus* | Observation-Intervention | Mundolkiri | Roveak | Cow | *P. vivax* | 5:00 PM |
| *A. indefinitus* | Observation-Intervention | Mundolkiri | Roveak | Cow | *P. vivax* | 7:00 PM |
| *A. indefinitus* | Observation-Intervention | Mundolkiri | Roveak | Cow | *P. vivax* | 7:00 PM |
| *A. indefinitus* | Observation-Intervention | Mundolkiri | Roveak | Cow | *P. vivax* | 12:00 AM |
| *A. indefinitus* | Observation-Intervention | Mundolkiri | Roveak | Cow | *P. vivax* | 12:00 AM |
| *A. indefinitus* | Observation-Intervention | Mundolkiri | Roveak | Cow | *P. vivax* | 12:00 AM |
| *A. indefinitus* | Observation-Intervention | Mundolkiri | Roveak | Cow | *P. vivax* | 12:00 AM |
| *A. indefinitus* | Observation-Intervention | Mundolkiri | Roveak | Cow | *P. vivax* | 4:00 AM |


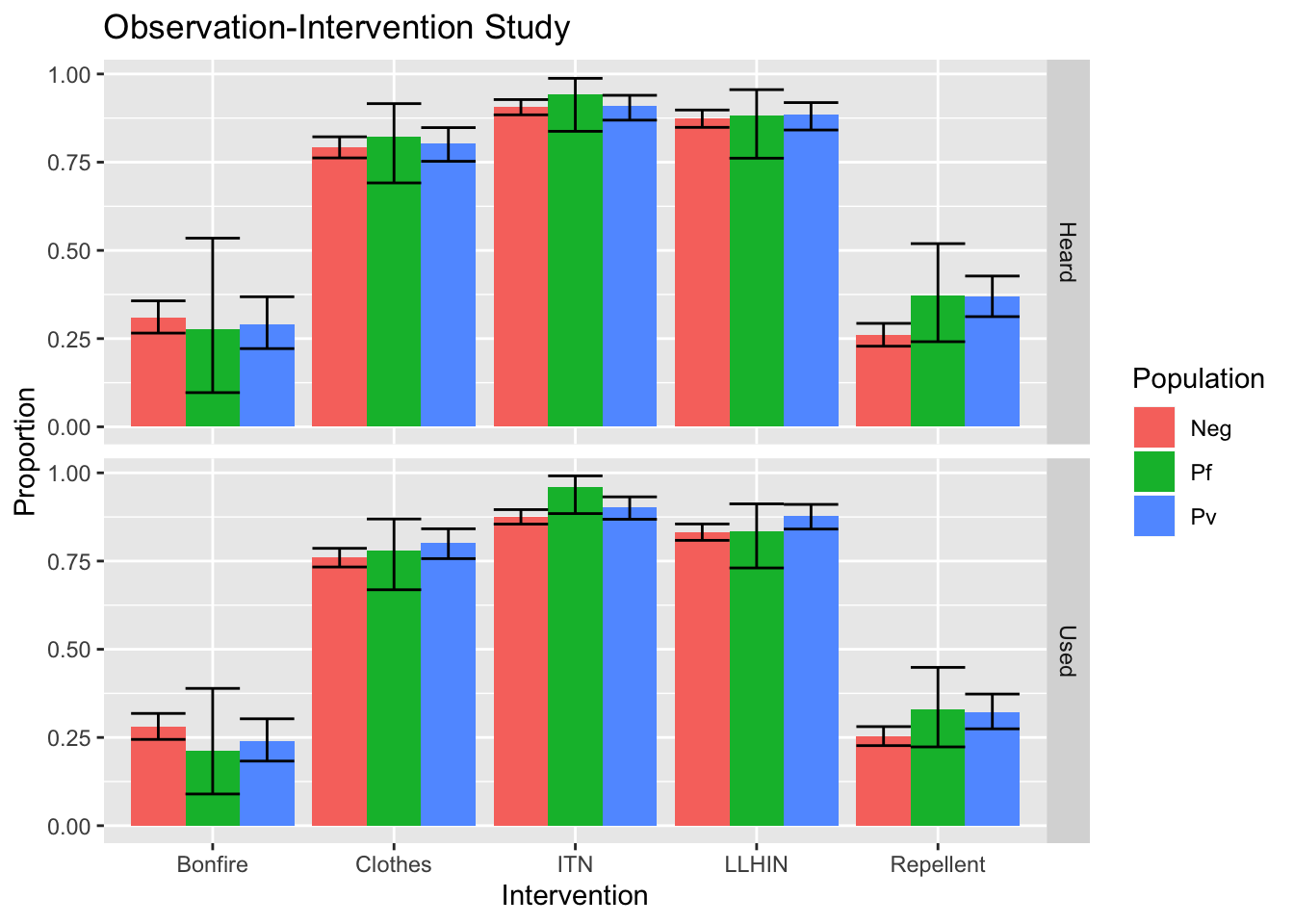


Fig S3: Proportion of individuals with *P. falciparum* infection, *P. vivax* infection, and no malaria infection (as measured by PCR) who have ever heard of or used the listed vector control measures, in the observation-intervention study.


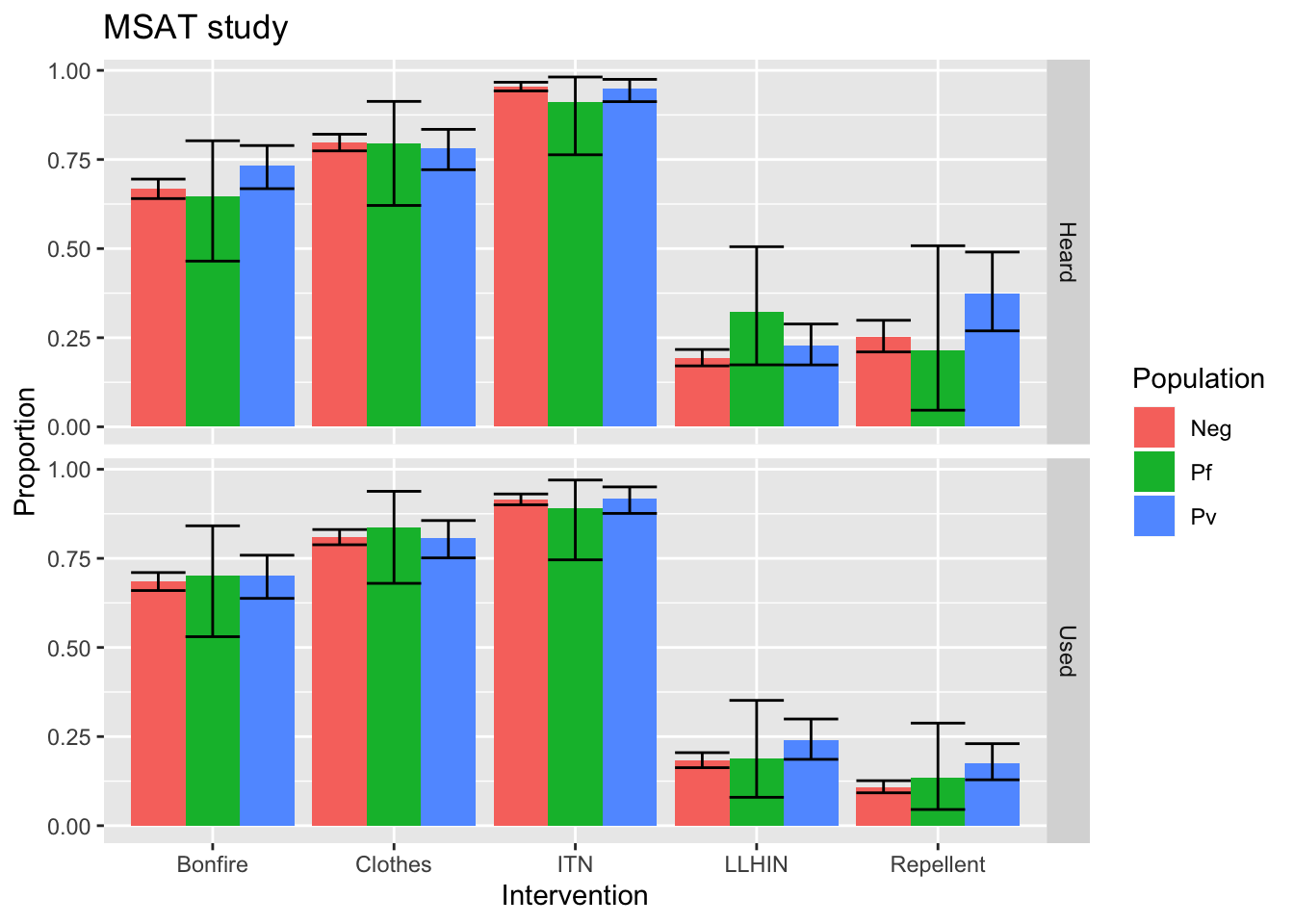


Fig S4: Proportion of individuals with *P. falciparum* infection, *P. vivax* infection, and no malaria infection (as measured by PCR) who have ever heard of or used the listed vector control measures, in the MSAT study.
